# Prevention of Heat-Stroke

**Published:** 1881-09

**Authors:** 


### Selections.

### PREVENTION OF HEAT-STROKE.

The *British Medical Journal* gives an analysis, from the Army Sanitary Regulations of Germany (Kriegs-Sanitats-Ordnung), of the directions concerning the prevention of heat stroke among troops on the march. These directions have evidently been framed from experience and a careful consideration of the whole subject and its bearings, and though intended specially for military men are equally instructive to civilian physicians. The instructions are given under the five following sectional headings: General Remarks, Causes, Symptoms, Prevention, and Treatment.

*General Remarks.*—The attention and activity of surgeons ought to increase in proportion to the efforts demanded from soldiers during the march, especially if the march is taking place in a heated atmosphere, for it is then that cases of heat-stroke are imminent. As a matter of diagnosis heat-stroke is to be distinguished from sun-stroke. The effects of sun-stroke, which, in tropical climates, may induce meningitis and encephalitis, in European climates are limited to inflammatory conditions and blistering of the skin of the parts of the body exposed to the solar rays. Heat-stroke is a far more serious and dangerous condition. It is a malady which, in certain circumstances, under the influence of great heat, with a clouded sky as well as in clear weather, is developed very rapidly, and one which, unless opportune assistance is afforded, may lead to death, sometimes within a few hours, sometimes after days have elapsed. Every officer directing the movements of troops ought to understand the causes of heat-stroke, as the disease is a preventable one.

*Causes.*—Among the conditions which favor the production of heat-stroke are the following: (*a.*) An elevated external temperature, especially when the air is muggy; that is, when it is charged with a large quantity of watery vapor, and there is no wind, so that perspiration does not evaporate, and, consequently, the natural process for cooling the body is interrupted; (*b.*) Physical efforts, which induce an elevation of the internal temperature of the body; (*c.*) Deficiency of water, that is, the absence of one of the most natural of the internal means of bodily refrigeration, on which depends the preservation of a due proportion of water in the blood and the supply of materials necessary for transpiration; and (*d.*) Insufficient ventilation of the column of men on the march.

Besides the above causes, there are a number of influences, the concurrence of which favor the invasion of heat-stroke. Such are: a want of the habit of marching; a constitution too weak for much exertion; anterior fatigue or illness; insufficient sleep; excesses; and, above all, indulgence in alcoholic drink.

It may be readily understood that at a time of great heat and under other external circumstances the appointed means of regulating the temperature of the body, and maintaining it at a constantly even degree, become insufficient for troops marching on foot in close ranks, particularly when the marches are made, as they often are, during the warmest hours of the day. The sweat, in close weather, cannot evaporate sufficiently; it collects on the face, on the chest, and flows over the body. This is the first refrigerant which fails. If, further, the soldier has not the means of drinking frequently to cool the body, and restore to the blood the water abstracted from it by transpiration, if the production of heat continues as a result of muscular efforts of the march, it may happen, on the one hand, that the blood becomes thickened, and the tissues dry and impaired; on the other hand, that the bodily temperature attains a degree dangerous to life. The man is then struck by heat-stroke. The two other regulators of the temperature, respiration and circulation, are not then sufficient for cooling the body; they become disordered under the influence of the extreme heat of the bodily economy, and in the end paralysis of the heart ensues.

To these causes must be added, when soldiers march in close order in calm weather, the vitiation of the air in which the column moves. Every one who has marched with troops knows what a fetid condition the air attains in warm weather, when there is not a strong wind to move it fast enough and renew it sufficiently.

Among cavalry soldiers heat-stroke happens more rarely than with infantry, because the muscular efforts, and, therefore, the elevation of temperature, are less with them, and because they move at wider distances apart than men marching on foot.

*Symptoms.*—The premonitory symptoms of heat stroke are the following: The perspiration is abundant on the surface of the body, yet the pulse is rapid, the respiration hurried, and the heart beats violently. The soldier feels his head hot, as well as his skin; he has a sense of constriction at the chest; a feeling of faintness; his tongue is dry, his hands swollen, and his face turgid or scarlet; his limbs totter. If at this time a man falls out of the ranks, he will generally soon be restored on being relieved from the pressure of his uniforn and accoutrements, by exposure to fresh air, by having some water given him to drink, and by applying a wet handkerchief to his head and chest. But if this help be not afforded and he drags himself painfully onwards, the transpiration becomes exhausted, the skin becomes dry, the lips glued together, the heart beats more feebly and more quickly, the respiration becomes more superficial, and at last the man loses sense and falls, sometimes as if in a fit, sometimes even with the signs of having became suddenly insane. When the condition has reached this degree, death is certain, unless help be afforded. Occasionally, the group of symptoms of heat-stroke are different, and depend on congestive determinations towards the brain, the lungs, or other organs, according to circumstances.

*Prevention.*—Soldiers the most habituated to marching cannot prevent their bodies from becoming overheated under certain circumstances. The natural remedy for this condition is drinking water. The belief that swallowing water is hurtful when the body is heated, arises from prejudice. Of course, some precaution must be used if the liquid is very cold. It is important not to swallow a large quantity at a draught; it is better to drink more often and less at a time, and to mix with the water a little vinegar, tea, or coffee. To wait long that the body may become cool is useless; a few minutes suffice. The wooden water-bottles with narrow throats, which soldiers usually carry, answer all the purposes required. If circumstances permit, it is important at periods when, after the morning, the temperature reaches 77° F. in the shade, to arrange the march so that part of it may be accomplished by eight or nine o'clock A.M., the men reaching a halting place at that time; the rest of the march can be taken late in the afternoon or evening. Before quitting a cantonment or halting place, it should be seen that the water-bottles are filled. Spirits should be avoided. When the soil is sandy, or the roads dusty, it is well to march in as open rank as possible. Halts should be multiplied and prolonged in airy places as far as practicable. Other points have to be attended to in time of war, but need not be described now.

*Treatment.* When a man is attacked by heat-stroke, he should, pending the arrival of a surgeon, be carried to as cool and shady a place as possible; his clothes and accoutrements should be unfastened; his head raised; and care should be taken that he is surrounded as little as possible by people, who would prevent the access of pure air. Cold should be applied, by means of wet towels, to the face and chest, and if this be not sufficient, cold affusions, especially over the trunk, should be had recourse to. The man should be made to swallow water freely, only a small quantity being given at a time. When respiration appears to be nearly arrested, artificial respiration should be proceeded with in the usual way. This should be continued if natural respiration fails to be quickly re-established, until the arrival of the medical officer. Air should be constantly conducted to the sick man while these means of restoration are in progress. Friction of the palms of the hands, and of the soles of the feet, and other means of stimulating the circulation, are advantageous. Drowsiness should excite mistrust; sleep should be watched with much care. In case of hemorrhage occurring, the man's comrades should abstain from all stimulating or exciting applications, and merely attend to the general measures above mentioned until the arrival of the medical officer, who will then give whatever directions the particular circumstances of the case may call for.

